# Spectral and Informational Analysis of Temperature and Chemical Composition of Solfatara Fumaroles (Campi Flegrei, Italy)

**DOI:** 10.3390/e23050593

**Published:** 2021-05-11

**Authors:** Simona Tripaldi, Luciano Telesca, Michele Lovallo

**Affiliations:** 1 Dipartimento di Scienze della Terra e Geoambientali, Università degli Studi di Bari Aldo Moro, Campus Universitario, via Orabona 4, 70125 Bari, Italy; 2 Institute of Methodologies for Environmental Analysis, National Research Council, 85050 Tito, Italy; 3 Agenzia Regionale per la Protezione dell’Ambiente della Basilicata, 85100 Potenza, Italy

**Keywords:** spectral analysis, Fisher–Shannon analysis, temperature and chemical composition, fumaroles, Campi Flegrei caldera

## Abstract

Temperature and composition at fumaroles are controlled by several volcanic and exogenous processes that operate on various time-space scales. Here, we analyze fluctuations of temperature and chemical composition recorded at fumarolic vents in Solfatara (Campi Flegrei caldera, Italy) from December 1997 to December 2015, in order to better understand source(s) and driving processes. Applying the singular spectral analysis, we found that the trends explain the great part of the variance of the geochemical series but not of the temperature series. On the other hand, a common source, also shared by other geo-indicators (ground deformation, seismicity, hydrogeological and meteorological data), seems to be linked with the oscillatory structure of the investigated signals. The informational characteristics of temperature and geochemical compositions, analyzed by using the Fisher–Shannon method, appear to be a sort of fingerprint of the different periodic structure. In fact, the oscillatory components were characterized by a wide range of significant periodicities nearly equally powerful that show a higher degree of entropy, indicating that changes are influenced by overlapped processes occurring at different scales with a rather similar intensity. The present study represents an advancement in the understanding of the dominant driving mechanisms of volcanic signals at fumaroles that might be also valid for other volcanic areas.

## 1. Introduction

Changes in temperature and composition at fumaroles are widely used to monitor volcanic activity aimed to surveillance [1,2,3]. These observations shed light on complex endogenous processes and offer the opportunity to reveal driving forces that occur at different time-space scales. However, along the path from the deep-rooted magmatic component to surface, many processes (e.g., magmatic volatile solubility, scrubbing, permeability) control the timing and the amplitude of temperature and composition changes. From the early precursory to post eruption stages, the geochemical signature and thus the monitoring activity is site-specific and requires case by case evaluations [4].

There are several cases in which the analysis of temperature and composition in fumaroles was useful to get insight into the dynamical processes taking place in volcanoes. The chemical and isotopic composition of Cumbal fumaroles (Colombia) were interpreted as reflecting a long period dominated by magmatic volatile input, which ends with an increased hydrothermal signature; the differences among discharging sites suggest differences in flow paths along which ascending gases may or may not be quenched by the hydrothermal system and/or meteoric water [5]. At the Merapi volcano (Indonesia), variations in the composition and temperature of fumarolic emissions were found to be related to atmospheric pressure and higher water concentrations formed by intensive rainfall (> 0.4 mm/5 min) [6], along with a correlation between temperature and a certain type of seismic activity (high-frequency seismic cluster and ultra-long period signal) [7]. At the Stromboli volcano, long-term (months to years) and short-term (hours to days) changes in soil temperature fumaroles were typical of crustal-driven and meteorological seasonal phenomena respectively, while abrupt changes were likely informative of precursors of explosive eruptive events [8]. The study of fumarole gas compositions and melt inclusions at the Kawah Ijen volcano (Indonesia) suggested a two-stage history implying the existence of shallower dacitic magma reservoir and a degassing deeper mafic one, which supplies metals and is characterized by progressive breakdown of sulfide [9]. The time variation in the fumarolic compositions at the Kusatsu-Shirane volcano (Japan) suggest a close relation between activation of seismicity and the increase of magmatic components; the compositional difference among the fumarolic gas recorded at different sites have been interpreted as reflecting the existence of three hydrothermal reservoir and formed by distinct condensation mechanisms [10]. Radon monitoring series at the two sites of Campi Flegrei caldera (Italy) were analyzed, and the results were compared with the CO/CO_2_ ratio, CO_2_ concentration, fumarolic tremor, ground deformation and the cumulative number of days with earthquakes [11,12]; the well correlated time variation of the independent signals suggest a general intensification of volcanic crisis at the caldera and that the current unrest involves an area much larger than the one characterized by seismicity and intense hydrothermal activity.

**Figure 1:**
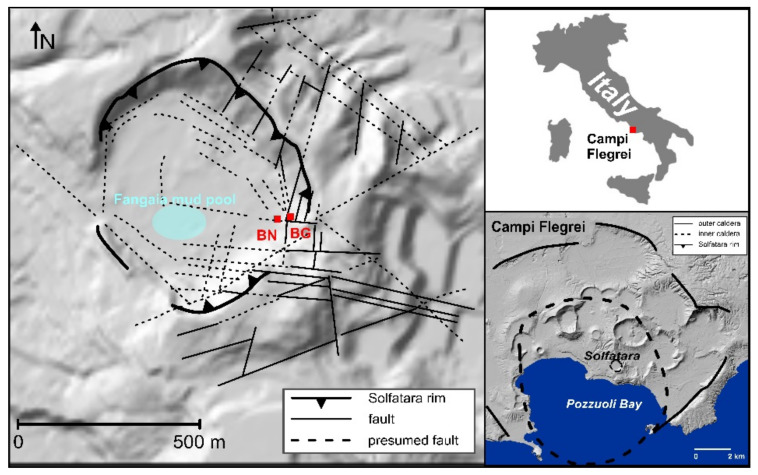
Location of Solfatara and Campi Flegrei; faults and presumed faults are reported from [16]; BN: Bocca Nuova fumarole; BG: Bocca Grande fumarole.

At Campi Flegrei caldera (CFc), geochemical, seismic and ground deformation measurements were carried out by the monitoring network, which was set up to manage the volcanic risk in a densely populated area. The CFc is a volcanic complex formed by two large explosive events (ca. 39 and ca. 15 ka) followed by numerous minor eruptions [13,14,15]. CFc is still active as testified by the last eruption (Monte Nuovo, 1538 AD), the bradyseismic episodes and the intense fumarolic and hot spring activity. The Solfatara is a maar-diatreme [16,17] located in the central sector of CFc (Figure 1), close to its most uplifted part (Pozzuoli area) and characterized by sustained hydrothermal activity. The crater is roughly elliptical in shape and is crossed by ring faults related to volcanic explosions and collapse of the crater center, regional faults striking mainly NW-SE and NE-SW and faults related to gravity instability of the volcanic rims [16]. This pervasive fault network allows the hydrothermal fluids to migrate and rise manifesting themselves in different form ad states. The surface expression of the Fangaia mud pool, the widespread soil diffuse degassing and the intense fumarolic activity reflect the lateral and vertical zoning of heterogeneity related to gas saturation, mixing between gas and condensed steam, fluid temperature and subsoil permeability [18,19,20,21]. The Fangaia mud pool is located in the most depressed zone of the crater (Figure 1), where the water table outcrops in a CO_2_-rich liquid domain and permeability is reduced by gas steam condensation and hydrothermal alteration [22]. The monitoring of diffuse volcanic degassing shows a significant expansion of the area releasing deep-sourced CO_2_ from 2003 to 2016; the amount of diffusively released CO_2_, recently assumed to be at least 2000–3000 t d^−1^, matches with typical values of persistently degassing active volcanoes [23]. Within the crater, the fumarolic activity is gathered in its southeastern part, at Bocca Grande and Bocca Nuova fumaroles (Figure 1). The study of the temperature, chemical and isotopic compositions of these fumaroles date to early 1980s. The main component of the fumaroles is H_2_O followed by CO_2_, H_2_S, N_2_, H_2_, CH_4_, He, Ar, and CO; temperatures at Bocca Grande and Bocca Nuova are well above the boiling point, respectively 161 ± 2.7 °C and 146.0 ± 2.0 °C [24,25]. Fumarolic chemical ratios studies, supported by physical–numerical simulations and geophysical investigations, have been carried out to get clues on the thermodynamic conditions and on the origin and evolution of the current unrest. Based on physical and volatile saturation models, it has been proposed that magma could be approaching the critical degassing pressure [26]. Accordingly, it was observed that the post 2000 background seismicity is increased at the same rate of the ground uplift and the concentration of the fumarolic gas specie more sensitive to temperature, likely because of repeated injections of magmatic gases from depth in accordance with a thermo-fluid-dynamic modeling [27]. Moreover, the current unrest is considered compatible with the ascent of CO_2_-rich hot gases from the deep (~8 km) magma chamber into the hydrothermal system and aquifers in nearly isenthalpic conditions, excluding both shallow arrival of new magma and steam condensation along the fumarolic flow path [28,29]. The current debate leaves room for further investigation needed for a better understanding of caldera dynamics.

In this paper, we analyze geochemical ratios and temperatures at Bocca Nuova and Bocca Grande fumaroles in the time span December 1997–December 2015. Our goal is to investigate the inner dynamics of the observed data and link it to the processes driving the observed changes. In this study we focus on the analysis (by using decompositional, spectral and informational statistical methodologies) of the oscillatory dynamics of the observed data, the detection of their periodicities (from monthly to years) and the identification of their volcanic or non-volcanic drivers.

## 2. Data

The dataset includes records of temperature and fluid compositions discharged at the main fumarolic vents in Solfatara, Bocca Grande and Bocca Nuova (Figure 1) from December 1997 to December 2015; the temperature and composition of the Solfatara fumaroles were obtained from literature [26]. We analyzed the monthly means series of temperature and CO/CO_2_, CO_2_/H_2_O, H_2_S/CO, He/CH_4_ and N_2_/He geochemical ratios; each series has a length of 217 samples. The percentage of missing data ranges from ~14% to ~17% at Bocca Grande and from ~15% to ~19% at Bocca Nuova.

The geochemical ratios are very important since they may provide information on specific volcanic processes. In fact, among fumarolic reactive gas species, CO is the most sensitive to temperature and CO/CO_2_ is considered the best gas-geothermometer in hydrothermal systems [30]. The CO_2_/H_2_O ratio reflects the magmatic component and it is a useful indicator of magma degassing [31]. Since hot, oxidized magmatic gases are rich in S species, almost converted in H_2_S when entering the hydrothermal system, H_2_S may reflect changes in the S-rich magmatic fluids input and variable steam separation [32]. In hydrothermal systems, the ratio between the He species of magmatic origin and the slow reactive specie CH_4_ (He/CH_4_) may provide information of the input of magmatic fluid in the hydrothermal system [25,33]; while the ratio between the inert gas species (N_2_/He) can reflect the parameters of the primary source (e.g., type of magma, pressure) of the fluids [34].

**Figure 2:**
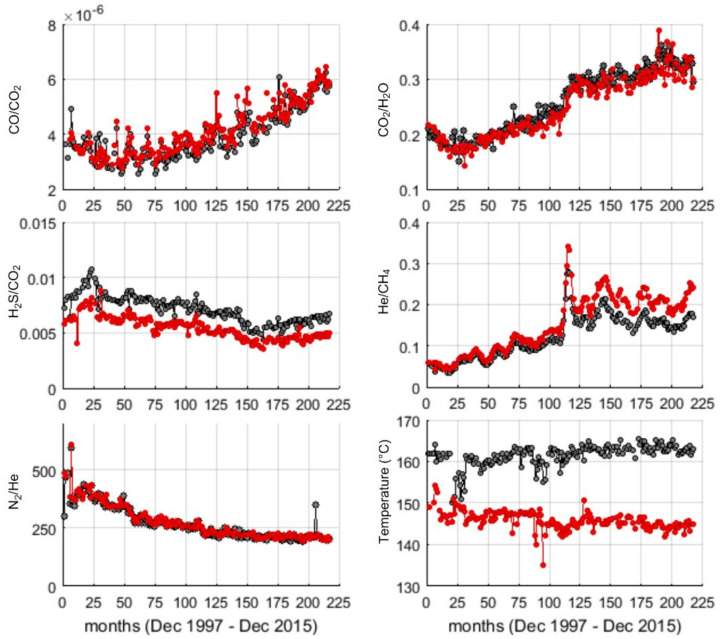
The monthly time series (black and red dotted lines respectively for Bocca Grande and Bocca Nuova).

All the geochemical ratios and temperature series are characterized by a quasi-oscillating component superimposed on a trend (Figure 2). CO/CO_2_, CO_2_/H_2_O and He/CH_4_ show an increasing trend, while H_2_S/CO_2_ and N_2_/He show a decreasing one. Comparing the same series related to the two different fumaroles, the CO/CO_2_, CO_2_/H_2_O and N_2_/He ratios can be almost overlapped; H_2_S/CO_2_ ratio and temperature recorded at Bocca Grande are slightly shifted up in comparison with the same observables recorded at Bocca Nuova. If until late 2007 (sample 120) He/CH_4_ ratio at both vents are almost overlapped after 2007 the ratio at Bocca Nuova is slightly increased (Figure 2).

## 3. Methods

### 3.1. Lomb–Scargle Periodogram

The Lomb–Scargle periodogram is used to estimate the power spectral density of unevenly sampled series, like those presenting gaps or missing data [35]. Considering a time series *x_k_*, where each value is observed at time *t_k_*, for *k* = 1,...,*N*, the Lomb–Scargle periodogram of the series *x_k_* is defined as follows:

(1) $$P_{LS}(f)=\frac{1}{2\sigma^{2}}\left\{\frac{{\left[\sum_{k=1}^{N}\left(x_{k}-\bar{x}\right)\cos\left(2\pi f\left(t_{k}-\tau \right)\right)\right]}^{2}}{\sum_{k=1}^{N}\cos^{2}\left(2\pi f\left(t_{k}-\tau \right)\right)}+\frac{{\left[\sum_{k=1}^{N}\left(x_{k}-\bar{x}\right)\sin\left(2\pi f\left(t_{k}-\tau \right)\right)\right]}^{2}}{\sum_{k=1}^{N}\sin^{2}\left(2\pi f\left(t_{k}-\tau \right)\right)}\right\}$$

where

(2) $$\bar{x}=\frac{1}{N}\sum_{k=1}^{N}x_{k}$$

and

(3) $$\sigma^{2}=\frac{1}{N-1}\sum_{k=1}^{N}{\left(x_{k}-\bar{x}\right)}^{2}$$

are respectively mean and variance of *x_k_*. The time offset τ is chosen as:

(4) $$\tan\left(4\pi f\tau \right)=\frac{\sum_{k=1}^{N}\sin\left(4\pi f\tau \right)}{\sum_{k=1}^{N}\cos\left(4\pi f\tau \right)}$$

It was demonstrated that a peak in the periodogram occurs at the same frequency which minimizes the sum of squares of the residuals of the fit of a sine wave to the data [36]. Due to the presence of data missing in the in the analyzed series, we used the Lomb-Scargle (LS) periodogram (we calculated the LS periodogram by using the Matlab built-in function plomb, https://it.mathworks.com/help/signal/ref/plomb.html accessed on 29 March 2021).

### 3.2. Singular Spectrum Analysis (SSA)

The singular spectrum analysis SSA [37] is a non-parametric method that extracts interpretable components such as slowly varying trends, periodic or quasi-periodic oscillations and structureless noise from short and apparently noisy time series [38]. The SSA is a principal components analysis (PCA) technique, where the input vectors comprise a time-series and phase-lagged copies of itself. The method basically consists into two main steps: decomposition and reconstruction. Let *y_i_* be a real-valued time series where *i* varies from 1 to *N* (length of the signal), for a lag M, the Toeplitz lagged correlation matrix expressed as:

(5) $$c_{j}=\frac{1}{N-j}\overset{N-j}{\underset{i=1}{\sum }}y_{i}y_{i+j},\;\;\;\;\;0\leq j\leq M$$

provides the eigenvalues λ*_k_* and eigenvector *E_kj_* sorted in decreasing order of λ*_k_*, with *j* and *k* varying from 1 to *M*. 

The *k*th principal component is defined by:

(6) $$a_{ik}=\overset{M}{\underset{j=1}{\sum }}y_{i+j}E_{jk}\;\;\;\;\;\;\;\;\;0\leq i\leq N-M$$

The *k*th reconstructed component of the signal is then given by:

(7) $$r_{ik}=\frac{1}{M}\overset{M}{\underset{j=1}{\sum }}a_{i-j,k}E_{jk}\;\;\;\;\;\;\;\;\;\;\;\;\;M\leq i\leq N-M+1$$

Since λ*_k_* is the fraction of the total variance of the original series contained in the *k*th rik, sorting λ*_k_* in decreasing order make also the corresponding reconstructed components ordered by decreasing information about the original series [39]. For time series with missing data, Schoellhamer [39] modified the calculation of the lagged autocorrelation and principal components as follows:

(8) $${\bar{c}}_{j}=\frac{1}{N_{l}}\underset{l\leq N-j}{\sum }{\bar{y}}_{l}{\bar{y}}_{l+j}\;\;\;\;\;\;\;\;\;0\leq j\leq M-1$$

that ignores any pair of data points with a missing value for *N_l_* pairs with no missing data and provides the eigenvalues and the eigenvectors with no gaps. The computation of the *k*th principal component ignores missing data points. The reconstruction step is performed as with SSA.

### 3.3. Fisher–Shannon Method

The Fisher–Shannon (FS) method allows to analyze complex time series by jointly using the Fisher Information Measure (FIM) and the Shannon entropy (SE). The FIM and SE describe the probability density function of a series, respectively, at a local and global level [40], and are generally employed to study the complexity of non-stationary time series in terms of order and organization (FIM) and disorder and uncertainty (SE) [41]. The FIM and SE are calculated, as follows:

(9) $$\mathrm{FIM}=\int_{-\infty }^{+\infty }{\left(\frac{\partial }{\partial x}f(x)\right)}^{2}\frac{dx}{f(x)},$$

(10) $$\mathrm{SE}=-\int_{-\infty }^{+\infty }f_{X}(x)\log f_{X}\left(x\right)dx,$$

where *f*(*x*) is the probability density function of the series *x*. Because SE can also be negative, the exponential transformation of Shannon entropy is generally applied to obtain the so-called Shannon entropy power *N_X_* that is commonly utilized in statistical analysis:

(11) $$N_{X}=\frac{1}{2\pi e}e^{2H_{X}}.$$

According to the isoperimetric inequality $\mathrm{FIM}\cdot N_{X}\geq D$ [42], where *D* is the dimension of the space (1 in case of time series), the FIM and the *N_X_* are interrelated; the equality stands in case of Gaussian processes. Due to the isoperimetric inequality, a better description of the dynamics of a time series can be obtained by using jointly both the measures. It was also shown in Martin et al. (1999) [43] that FIM allowed for the detection of some non-stationary behavior in situations where the Shannon entropy showed a limited detection power. As the calculation of FIM and *N_X_* depends on the probability density function, attention has to be paid to its good estimation. In this study, we used the kernel-based approach to estimate *f*(*x*), which has been shown to have a better performance than the discrete-based approach in calculating the value of FIM and SE for the Gaussian distributed series [44]. The kernel-based approach for estimating the probability density function is based on the kernel density estimator technique [45,46]:

(12) $${\hat{f}}_{M}(x)=\frac{1}{Mb}\sum_{i=1}^{M}K\left(\frac{x-x_{i}}{b}\right),$$

where *b* refers to the bandwidth, *M* represents the number of data, and *K*(*u*) is a continuous non-negative and symmetric kernel function that satisfies the following two conditions:

(13) $$K\left(u\right)\geq 0\;\mathrm{and}\;\int_{-\infty }^{+\infty }K(u)du=1,$$

The estimation of *f*(*x*) uses an optimized integrated method [43] that is based on Troudi et al.’s [47] and Raykar and Duraiswami’s [48] algorithms, where a Gaussian kernel with zero mean and unit variance is adopted:

(14) $${\hat{f}}_{M}(x)=\frac{1}{M\sqrt{2\pi b^{2}}}\sum_{i=1}^{M}e^{-\frac{{\left(x-x_{i}\right)}^{2}}{2b^{2}}}$$

The isoperimetric inequality enables the application of the Fisher–Shannon (FS) information plane to explore the dynamics of a series [49], in which the coordinate axes are *N_X_* and FIM. For scalar signals, the line FIM·*N_X_* = 1 divides the FS information plane into two parts, and each signal is represented by a point that lies exclusively in the half-space of FIM·*N_X_* > 1.

## 4. Results

**Figure 3:**
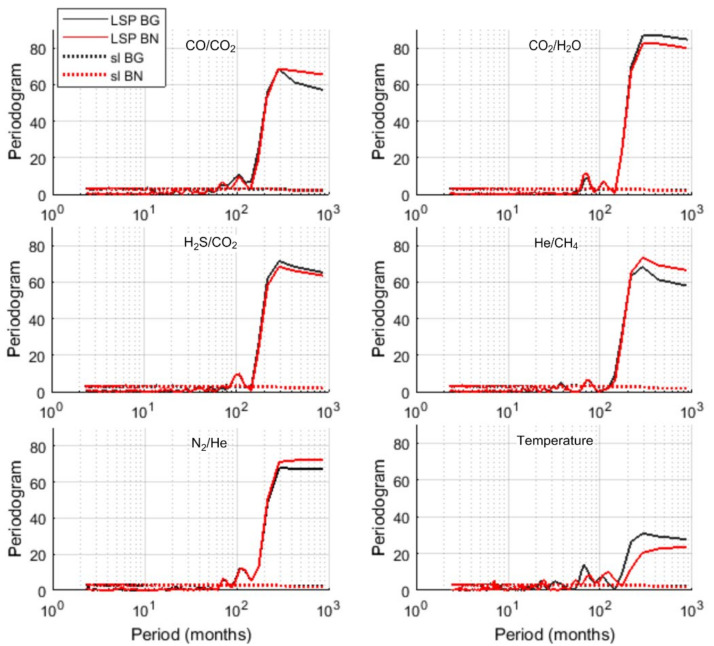
Lomb–Scargle periodogram (LSP) of the time series along with the significance levels (sl).

We applied the Lomb–Scargle periodogram (LSP) to investigate the spectral properties of the investigated time series (Figure 3). For each spectrum we also calculated its 95% confidence curve (red), obtained as the 95th percentile of the distribution at each frequency of the values of the LSP of 1000 surrogates generated as random shuffles of the original series; thus, all the LSP peaks of the original series above the 95% confidence curve can be considered significantly not random. This preliminary analysis shows that for most of the time series the spectral content is mainly concentrated in the region of the very low frequencies that suggests the dominance of the trend. The dominance of the trend does not make easy the detection of the oscillatory components.

In order to better explore the time dynamics of the investigated series, we applied the SSA. The SSA requires the selection of a proper window length M. For relatively short series modulated by an oscillation of period T, the window length M should be proportional to T [50] and satisfying the Khan and Poskitt’s criterium [51]. In our case, we selected M = 36, that allows the extraction of the oscillatory components without losing much information on long-term fluctuations. We applied the Schoellhamer’s SSA algorithm [39] with a fraction of missing data points f = 0.5 within the window size M = 36. Thus, each series was decomposed into 36 independent components, the first one (RC1) representing the trend.

The contribution of the first component (RC1) to the variance of each time series, given by its corresponding eigenvalue, is reported in Table 1. Unlike temperature series, the first reconstructed components of the geochemical ratios explain the great part of variance of the series.

**Figure 4:**
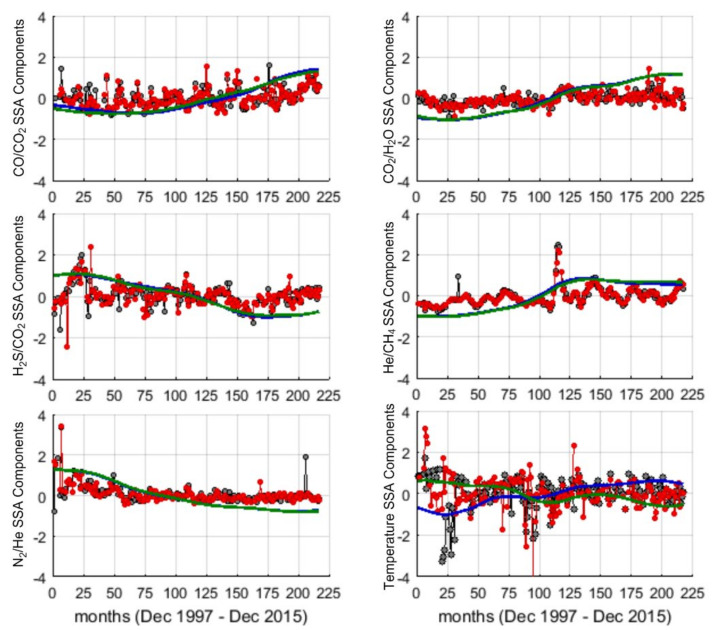
RC1 (blue and green lines respectively for Bocca Grande and Bocca Nuova); RS (black and red dotted lines respectively for Bocca Grande and Bocca Nuova).

Figure 4 shows for each series the trend (RC1) and the residual (RS), given by removing the trend from the original series. In both the vents, for CO/CO_2_, CO_2_/H_2_O the trend has increasing behavior; for He/CH_4_ after a slight increase until early 2008 (125th month) the trend seems to be almost stable; for H_2_S/CO_2_ and N_2_/He the trend is characterized by a decreasing behavior. The trend of temperature at Bocca Grande and Bocca Nuova shows opposite behaviors. For all the series the RS is modulated by an oscillatory behavior.

**Figure 5:**
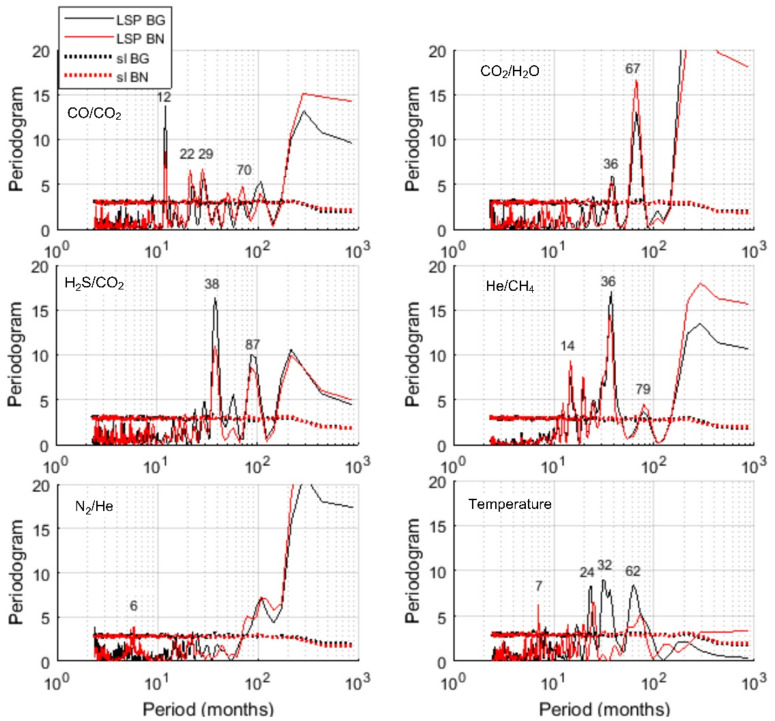
Lomb–Scargle periodogram (LSP) of the residual series (RS) along with the significance curves (dotted lines) levels (sl) for Bocca Grande (BG) and Bocca Nuova (BN).

Figure 5 shows the LSP along with the 95% significance levels of the RSs. CO/CO_2_, He/CH_4_ ratios display significant periodicities ranging from 12 to 79 months, at both Bocca Grande and Bocca Nuova vents. The RS of temperature recorded at Bocca Nuova shows the periodicity of 7 months, absent in the RS of temperature recorded at Bocca Grande; while the 32-months periodicity is found only in Bocca Grande. The RS of CO_2_/H_2_O and H_2_S/CO_2_ are characterized by the presence of medium to long term periodicities ranging from 38 to 87 months. The LSP of the RS of N_2_/He ratio is very different from the others: the periodogram occupies mainly the low frequency band and only very short (2.5 and 6 months) periodicities are significant.

**Figure 6:**
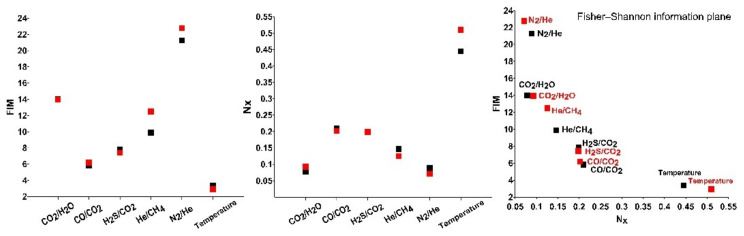
FIM, Nx and FS information plane of the residuals for Bocca Grande (black) and Bocca Nuova (red).

The order/disorder pattern of the RSs (Figure 6) shows that the dominance of order or organization in the series is not strictly linked with the vent; for instance, some series recorded at Bocca Grande (N_2_/He, He/CH_4_) are characterized by larger disorder degree than those recorded at Bocca Nuova. However, the FS plane shows that the series are almost aligned, with N_2_/He occupying the top left part of the plane and temperature occupying the right bottom part of the plane; this indicates that N_2_/He of the residual series in both the vents is characterized by higher organization and lower disorder, while the temperature by larger disorder and lower organization. The other series are placed in the middle between the positions of N_2_/He and temperature.

## 5. Discussion and Conclusions

The trend that represents the very low frequency dynamics of the series, was separated by the oscillatory residual by the SSA. Concerning the temperature, the trend does not account for most of the variability of the original series (30–39%), and this indicates that short to medium term processes mostly govern the time dynamics of temperature. The trend of the geochemical ratios, instead, accounts for most of their variability (from 71% to 87%), indicating a clear dominance of very low frequency fluctuations. This result suggests that the geochemical ratios mainly reflect the direction of deep large-scale processes, which may have magmatic or hydrothermal origin (e.g., interaction between the input of magmatic gases and the recharge-discharge dynamics of the reservoir). This result is in agreement with [52], who developed numerical models aimed at evaluating the dynamics of the coupled water-magmatic system at CFc; the authors found decennial cycles, likely associated to the heating at depth interacting with the recharge-discharge dynamics of the reservoir where faults and permeability play a crucial role. Consistently, the pattern of fumarolic effluents and geophysical signals at CFc recorded during the period 2000–2008 was found to be interpretable as increment of the relative amount of magmatic fluids hosted by the hydrothermal system favored by the opening of an easy ascent pathway toward the brittle domain [24]. The pattern of geochemical records after 2005 was found to be consistent with a state of unrest related to the hydrothermal activity [53]. The long-term decreasing trend of relative velocity variations during 2010–2014, derived by noise-based seismic monitoring, has been interpreted (at the light of the increased release of H2O-rich magmatic gas) as a gradual heating of the hydrothermal system and compatible with the upper 4 km crustal rheological change from elastic to plastic since the 1982–1984 bradyseismic crisis inferred by the study of temporal and spatial evolution of the 1982–2014 seismicity [54,55].

The analysis of the RSs has revealed how rich is the time dynamics of the investigated signals. The LSP of CO_2_/H_2_O and H_2_S/CO_2_ shows similar periodic structure with most of the power concentrated at medium-long periods (36–38 months for both and 67 and 87 months respectively for CO_2_/H_2_O and H_2_S/CO_2_). CO/CO_2_, He/CH_4_ and temperature share a more complex periodic structure, characterized by the presence of several significant periodicities from 7 to 79 months. Since magmatic processes can have characteristic periods of the order of several months [56], CO_2_/H_2_O and H_2_S/CO_2_ may be considered to track the timing of deep magmatic driving processes; while CO/CO_2_, He/CH_4_ and temperature could allow the recognition of recurrent processes occurring in the hydrothermal system and that are also influenced by non-magmatic processes.

The periodicities of 62–87 months commonly found in the series may reflect processes that drive ground deformation episodes widely reported in the CFc literature [57,58]. In fact, after the bradyseismic crisis occurred in 1982–84, the pattern of the ground displacement is made up of a deflation pattern which reverses, since 2005, in an inflation pattern that is still ongoing. Small-scale episodes of faster uplift, named mini-uplift, are superimposed on the general trend and have a recurrent time of 5–6 years [29,59]. Ground uplift episodes have been ascribed to hydrothermal circulation, to magma intrusion at shallow depth, or to repeated injection of magmatic fluids into the hydrothermal system [25,60,61,62]. Chiodini et al. (2003) [61] analyzed correlations between fumarolic ratios with ground deformation and performed numerical modeling accounting for periodic injections of hot CO_2_-rich fluids at the base of the hydrothermal system; the good fitting between observed and simulated CO_2_/H_2_O ratio together with the good pattern correspondence to the observed uplift episodes suggested that periodic intense magmatic degassing may origin both geochemical ratios’ changes and ground deformation [61]. The periodicity of about 67 months retrieved in CO2/H2O is in good agreement with the observational evidence of recurrence of mini-uplift episodes and with the findings of the studies mentioned above. Similar periodicities are also present in residuals of CO/ CO_2_, He/CH_4_ and temperature, but less powerful.

As widely reported, the geochemical and geophysical signatures at CFc also depends on rocks properties, as shown by thermo-fluid dynamical modeling at CFc. Indeed, several numerical models show that heterogeneities in hydrological and mechanical properties may influence the timing and the amplitude of signals’ changes through time [63,64,65]. The interplay between magmatic gas raising from depth and the recharge-discharge dynamics of the reservoir gives rise to periodic geophysical and chemical signatures [52]. Within this context, it is noteworthy the relationship found between the NAO (North Atlantic Oscillation) index and the groundwater recharge of carbonate karst aquifers in Campania region analyzing data from 1921 to 2010 [66]; in particular, the main periodogram peaks of the regional normalized indexes of precipitation and spring discharges and those of NAO well match at 2–3, 5 and 45 years. The periodicities in the range of 29–38 months of CO_2_/H_2_O, H_2_S/CO_2_, CO/CO_2_, He/CH_4_ residuals at both Bocca Grande and Bocca Nuova and of temperature at Bocca Grande, well match with those of NAO index periodicities from 2 to 3 years found in Campania region. This result could indicate that the geodynamics of fumaroles may be influenced by hydrological cycle of large regional aquifers, which in turn may be linked to NAO. Although it is reasonable to assume a relationship between the fumarole’s activity and groundwater recharge processes, this finding would deserve further investigations.

Short term periodicities found in CO/CO_2_, He/CH_4_ and temperature residuals indicates that these variables may reflect the influence of seasonal external forces. Short term periodicities have also been observed in seismic activity at CFc. The analysis of earthquakes occurred in the 2005–2016 observational interval, showed a cyclic behavior of the clustered seismicity ranging from semidiurnal to annual [67]. Notably, the authors find a correlation with rainfall series, observing a major occurrence of energetic swarm in the wet season. The possible influence of seasonal rainfalls on velocity variation derived by seismic noise monitoring at CFc from January 2010 to December 2014 has been also considered [54]. As reported by the authors, the velocity variation annual periodicity may be ascribed to changes of the permeability of the shallowest crust influenced by rainfalls. Moreover, by applying the independent component analysis technique to time series recorded at 16 GPS stations during the period 2001–2007, periodicities up to one year associable to atmospheric and oceanic loading processes were found [68]. Thus, it is reasonable to consider temperature, CO/CO_2_, He/CH_4_ as most reflecting the effects of exogenous processes.

The temperature at the two fumaroles (Bocca Grande and Bocca Nuova) shows different trends; although the two fumaroles are very close (~25 m apart), results indicate warming-up trend at Bocca Grande and a cooling trend Bocca Nuova. Furthermore, assuming that long to medium term periodicities are related to deep-medium rooted processes and that short-term periodicities are related to shallow processes, Bocca Grande would better reflect magmatic and hydrothermal processes interacting with the deep ground water recharge. In fact, in a recent study, it was shown that the different behavior of the trend could be due to the different geometry/properties of the fumaroles structure and inherent fluid flow; the cooling trend observed at Bocca Nuova could be produced by the mixing between gas and condensed steam, while Bocca Grande seems not affected by shallow water mixing [20].

The results obtained by applying the Fisher–Shannon method shed light on the relationship between organization/uncertainty of the series and the scales of the different processes governing their dynamics. As a general pattern, we observe that the entropy of the series, which informs about the uncertainty and loss of order in the series, seems to be correlated with the numerosity of the modes (significant peaks in the LSP) that characterize each series (Figure 5 and Figure 6). The LSP of the residual of N_2_/He is mainly concentrated in the very low frequency region, characterized by a single dominant mode of variability, and thus the best organized. CO_2_/H_2_O, He/CH_4_ and H_2_S/CO_2_ show a few significant medium-long periods (36–78 months), thus displaying higher degree of organization than CO/CO_2_ and temperature, which are, instead, characterized by a wide range of significative periodicities (from short to long) nearly equally powerful. In other words, CO/CO_2_ and temperature residuals seem concomitantly affected by different processes with different scales and thus their dynamics is featured by a higher entropy and a greater uncertainty. The two vents Bocca Grande and Bocca Nuova show the main difference in the behavior of temperature residuals: at Bocca Nuova, whose temperature residual is found to be more influenced by short term processes, it is characterized by a higher degree of disorder, likely indicating that shallower processes taking place at Bocca Nuova make its residual temperature more unpredictable.

In this study the combined approach based on the spectral and informational analysis contributes to the advancement of the knowledge of the dominant driving mechanisms underlying the time dynamics of volcanic on signals recorded at fumaroles that might be also be extended to other different volcanic settings.

## Figures and Tables

**Table 1:** Percentage contribution of the first component (RC1) to the variance of each time series.

	CO/CO_2_	CO_2_/H_2_O	H_2_S/CO_2_	He/CH_4_	N_2_/He	Temperature
RC1 BG	73%	87%	75%	72%	71%	39%
RC1 BN	74%	86%	72%	76%	73%	30%
